# Comparison of Results from Inpatient and Outpatient Treatment of Tuberculosis in Republic of Macedonia

**DOI:** 10.3889/oamjms.2015.050

**Published:** 2015-04-30

**Authors:** Ljiljana Simonovska, Biljana Ilievska-Popovska

**Affiliations:** *Institute for Lung Diseases and Tuberculosis, Faculty of Medicine, Ss Cyril and Methodius University of Skopje, Skopje, Republic of Macedonia*

**Keywords:** tuberculosis, outcome, sputum conversion, cured, died, Republic of Macedonia

## Abstract

**BACKGROUND:**

The successful treatment of patients with active tuberculosis is one of the priorities in the Tuberculosis Control Programs.

**AIM:**

The aim was to establish whether there was a statistically significant difference in the treatment outcome in patients with tuberculosis who began their initial treatment phase and/or pursued it as inpatient, as opposed to patients with tuberculosis who underwent their entire treatment regime as outpatient. Moreover, our goal was to determine whether there is a statistically significant difference in the outcome from the treatment between patients with tuberculosis who were hospitalized up to one month, two months, or more than two months.

**MATERIALS AND METHOD:**

The study includes 355 patients, divided into two groups. The first group, which consists of 219 patients, began their initial treatment phase as inpatient, and then they continued the treatment as outpatient. The second group, 136 patients, underwent their entire treatment as outpatient. The treatment outcome is determined with every patient (cured, treatment completed, treatment default, treatment failed, died, treatment in progress). For the statistical data analysis and for establishing the significance of the findings regarding the differences between the two groups we used the Pearson Chi-Square Test and the Yates Corrected Test.

**RESULTS:**

The statistical analysis with the Pearson Chi-Square Test showed that the treatment outcome does not significantly depend on the model of treatment (p = 0.31). The statistical data analysis showed that there is no statistically significant difference in the achievement of conversion of the bacterial result of the sputum at the end of the initial phase of treatment regarding the studied groups (p = 0.89). The statistical data analysis showed that the length of inpatient treatment affects the outcome of the treatment and that the difference is statistically highly significant (p < 0.00005).

**CONCLUSION:**

There are no statistically significant differences in the sputum conversion and the treatment outcome among inpatient/outpatient with tuberculosis or outpatient only; however, the length of inpatient treatment is statistically significant regarding its effect on the treatment outcome.

## Introduction

The successful treatment of patients with active tuberculosis is one of the priorities in the Tuberculosis Control Programs. The treatment guidelines suggest the target of 85% successfully treated cases of tuberculosis. In 1994 the World Health Organization made an estimation of the rate of success regarding the treated patients, which showed that results vary: a 77% success rate where the directly observed therapy (DOT) is used and only 41% in countries where DOT is not used.

In terms of the inpatient treatment, there are no conclusive findings on whether it gets better results in patients with tuberculosis [1]. According to numerous studies there is higher risk of intrahospital transmission of the infection [2], and also cause a greater socio-economic loss in patients and their families [3-6].

The American Thoracic Society recommends that priority be given to the outpatient treatment model for treatment of patients with tuberculosis, and that the infectiveness does not constitute a reason for hospitalization [4]. The same recommendation is given by the World Health Organization (WHO). According to Caminero [7], reasons for hospitalization are: poor physical condition, complications associated with tuberculosis, drug’s side effects, social reasons, and application of second line anti TB- medication.

There are studies regarding the patients with multi-drug-resistant tuberculosis (MDR-TB). During their case revision regarding patients with MDR-TB who were inpatient or outpatient, Bassili and his associates [8] came to the conclusion that there is not a single category in terms of the outcome (successful treatment, failed treatment, stopped treatment or death) where the treatment model played any role

The aim was to establish whether there was a statistically significant difference in the treatment outcome in patients with tuberculosis who began their initial treatment phase and/or pursued it as inpatient, as opposed to patients with tuberculosis who underwent their entire treatment regime as outpatient. Moreover, our goal was to determine whether there is a statistically significant difference in the outcome from the treatment between patients with tuberculosis who were hospitalized up to one month, two months, or more than two months.

## Material and Methods

The study includes 355 patients, who were treated from tuberculosis, in 2012.

The patients were divided into two groups. The first group, which consists of 219 patients, began their initial treatment as inpatient, and then they continued the treatment as outpatient. The second group, 136 patients, underwent their entire treatment as outpatient.

Criteria for inclusion in the research were: All new and previously treated patients with pulmonary and extrapulmonary tuberculosis, who have received their results from the treatment. Criteria for exclusion from the research were: All new and previously treated patients with pulmonary and extrapulmonary tuberculosis, who have not received their results from the treatment. All patients were treated by essential anti-tuberculosis medication

The treatment outcome is determined with every patient according treatment outcome definitions recommended by WHO and National TB strategy (cured, treatment completed, treatment default, treatment failed, died, treatment in progress). For the statistical data analysis and for establishing the significance of the findings regarding the differences between the two groups we used the Pearson Chi-Square Test and the Yates Corrected Test.

## Results

Table 1 shows distribution of patients with tuberculosis according to age.

**Table 1:** Distribution of patients with tuberculosis according to age

Age Groups	0-14	15-24	25-34	35-44	45-54	55-64	>65	Total
Number of patients	29 (8.16%)	59 (16.61%)	54 (15.21%)	51 (14.36%)	60 (16.90%)	44 (12.39%)	58 (16.33%)	355 (100%)

According to gender, 214 (60.28 %) were males, and 141 (39.71 %) were females; a scale of 1.5:1 (M:F).

Table 2 shows the distribution of the treatment outcome (cured, treatment completed, treatment default, treatment failed, died, treatment in progress) according to the model of treatment. The statistical analysis with the Pearson Chi-Square Test showed that the treatment outcome does not significantly depend on the model of treatment (p = 0.31).

**Table 2:** Distribution of the treatment outcome according to the model of treatment

Treatment outcome	Model of treatment		Total
	Outpatient treatment	Inpatient/outpatient treatment	
Cured	50 (38.76%)	79 (36.07%)	129
Treatment Completed	66 (48.53%)	96 (43.84%)	162
Treatment default	6 (4.41%)	10 (4.57%)	16
Treatment failed	1 (0.74%)	2 (0.91%)	3
Died	5 (3.68%)	23 (10.5%)	28
Treatment in progress	8 (5.88%)	9 (4.11%)	17
Total	136	219	355

Pearson Chi-square: 5.95, df=5, p=0.31.

The statistical data analysis showed that there is no statistically significant difference in the achievement of conversion of the bacterial result at the end of the second month for the new cases, and the end of the third month for the previously treated cases, that are at the end of the initial phase of treatment regarding the group of inpatient/outpatient and the group of outpatient only (p = 0.89).

The results of achievement of conversion of the bacterial results at the end of the second and the third month according to the model of treatment are shown in Table 3.

**Table 3:** Achievement of conversion of the bacterial results at the end of the second and the third month according to the model of treatment

Microscopy results at the end of 2/3 month	Model of treatment		Total
	Outpatient treatment	Inpatient/outpatient treatment	
Negative	56 (96.55%)	86 (95.56%)	142
Positive	2 (3.45%)	4 (4.44%)	6
Total	58	90	148

Yates corrected = 0.02, df=1, p=0.89.

The statistical data analysis showed that the length of the inpatient treatment affects the outcome of the treatment and that the difference is statistically highly significant (p < 0.00005). The results are shown in Table 4.

**Table 4:** Distribution of the treatment outcome according to the length of inpatient treatment

Treatment outcome	Length of treatment			Total
	1-30 days	30-60 days	HaД 60 days	
Cured	13 (18,57%)	25 (32.89%)	41(56.16%)	79
Treatment completed	41(58.57%)	36 (47.37%)	19(26.03%)	96
Treatment default	4 (5.71%)	4 (5.26%)	2 (2.74%)	10
Treatment failed	1 (1.43%)	1 (1.32%)	0	2
Dead	11 (15.71%)	8 (10.53%)	4 (5.48%)	23
Treatment in progress	0	2 (2.63%)	7 (9.59%)	9
Total	70	76	73	219

Pearson Chi-square: 37.29, df=10, p=0.00005.

## Discussion

The successful treatment of patients with active tuberculosis is one of the priorities in the Tuberculosis Control Programs. The treatment guidelines suggest the target of 85% successfully treated cases of tuberculosis.

In terms of the inpatient treatment, there are no conclusive findings of whether it gets better results in patients with tuberculosis [1].

In our retrospective study which includes 355 patients, out of which 136 (38.3%) were outpatient during the two phases of the therapy regime, and 219 (61.2%) were inpatient and outpatient, the statistical analysis with the Pearson Chi-Square Test showed that the tuberculosis treatment outcome does not significantly depend on the model (manner) of treatment (p = 0.31).

The statistical data analysis using the Yates Corrected Test showed that there is not a significant difference in the achievement of conversion in the bacterial result, as a criterion for the treatment success at the end of the initial phase of the treatment in the group of outpatient compared to the group of inpatient/outpatient (p = 0.89).

The statistical treatment outcome data analysis using the Pearson Chi-Square Test in patients with different lengths of inpatient treatment in this research (during the period of up to one, two or more than two months) showed that the length of the inpatient treatment statistically had a significant impact on the results of the treatment (p < 0.00005).

The best treatment outcome was recognized in patients who received the longest inpatient treatment, i.e. longer than two months. 56% of the patients were cured, which can be attributed to the fact that the tuberculosis patients are a heterogeneous group of patients. Often there are grave forms of tuberculosis with present risk factors (alcoholism, drug addiction), or the patients fall under different categories, often followed by comorbid conditions, such as: diabetes, psychiatric disorders, cardiomiopathy, bronchitis, and a possible present resistance which is not the subject of this research. All these factors condition the length of the inpatient treatment in some patients (longer than two months) so that they could get the best therapy response.

The treatment completed as an outcome which is statistically significant usually is present in the group of inpatient treated up to one month. This outcome is common in patients who are smear negative, or have extrapulmonary tuberculosis. Since it is a case of smear negative tuberculosis, the one-month inpatient treatment is regarded as a sufficiently long period in order to achieve satisfactory results in this category of patients. Or, perhaps this category should be treated as outpatient only.

According to the statistical data analysis, the number of “treatment default” was not enormous (10/219), but the break in treatment most often happened to patient who was hospitalized for one or two months, and they cut the treatment after they leave the hospital during the therapy regime. Perhaps they had not understood seriously the recommendation to take their medications regularly. Perhaps they have returned to their old habits. Nevertheless, the breaks in treatment are reasons for the appearance of a resistant form of tuberculosis, and it is necessary to make efforts to reduce the number of these patients to a minimum.

Although the number of inpatient whose treatment failed is very small, still that number is statistically bigger among the patients who were treated in hospital up to two months compared to patients who were treated more than two months. Some of the predictive factors include the bad cooperation, i.e. the irregular medicine intake after the inpatient treatment.

According to Wobeser [1], causes for treatment failure are: alcoholism, drug abuse, and the possible drug resistance which is not a subject of this research.

In this study we have come to a realization that in 10% (23/219) of the inpatient, the result is death. However, according to the statistical data analysis using the Pearson Chi-Square Test it is concluded that statistically death occurs most often in cases where the inpatient treatment lasted up to one month.

In the study by Perrechi [9], which included 474 patients, out of which 166 continued the treatment as inpatient and 308 as outpatient, it was concluded that the death rate in the inpatient was statistically higher (23.5%) compared to the death rate in outpatient (2.6%). By using a multivariate analysis it was shown that the hospitalization of the patients was associated with grave forms of tuberculosis.

Our analysis also showed that the statistically most significant death rate in patients who were treated for up to one month is due to predictive factors like the grave forms of tuberculosis, comorbid conditions, risk factors, such as alcoholism, which causes weakening of the non specific immunity, and the toxic hepatitis, which initiates a change in the therapy regimes, i.e. a usage of less hepatotoxic drugs, but also less effective therapy regimes.

According to Wobeser [6] some of the predictive mortality factors are: patients older than 50, immunosuppressant therapy, HIV, manifestation of drug’s side effects and the present antituberculosis drugs resistance.

The “treatment in progress” as a treatment outcome in this research is present in a small number of cases among the inpatient/outpatient (9/219). However, this treatment outcome is statistically more present in patients who were hospitalized longer than two months. These patients receive an individual treatment regime which depends on various allergic and toxic manifestations. The individual regimes usually include less effective antituberculosis drugs, and that prolongs the treatment. The weaker therapy response and the presence of toxic and allergic manifestations are independent predictive factors for the prolongation of the hospital and the overall treatment in separate tuberculosis cases.

The analysis brings us to the conclusion that there are no statistically significant differences in the sputum conversion and the treatment outcome among patients with tuberculosis who were treated as inpatient/outpatient or as outpatient only; however, the length of the inpatient treatment is statistically significant regarding its effect on the treatment outcome.
